# Four-dimensional computed tomography detects dynamic three-dimensional pathologies of the wrist in patients with calcium pyrophosphate deposition disease

**DOI:** 10.3389/fmed.2023.1231667

**Published:** 2023-08-04

**Authors:** Sevtap Tugce Ulas, Louise Pochandke, Sarah Ohrndorf, Torsten Diekhoff, Katharina Ziegeler

**Affiliations:** ^1^Department of Radiology, Charité – Universitätsmedizin Berlin, Campus Mitte, Humboldt – Universität zu Berlin, Freie Universität Berlin, Berlin, Germany; ^2^Berlin Institute of Health at Charité – Universitätsmedizin Berlin, Berlin, Germany; ^3^Department of Rheumatology and Clinical Immunology, Charité – Universitätsmedizin Berlin, Campus Mitte, Humboldt – Universität zu Berlin, Freie Universität Berlin, Berlin, Germany

**Keywords:** computed tomography, CPP disease, wrist, 4D-CT, Arthropathy

## Abstract

**Objectives:**

Crystal deposits in ligaments of the wrist are typical findings in patients with calcium pyrophosphate deposition (CPPD) disease. CPPD crystals trigger inflammation and ultimately result in ligament tears with scapholunate (SL) advanced collapse (SLAC). This study aimed to investigate carpal instabilities in patients with CPPD using four-dimensional computed tomography (4D-CT) of the wrist.

**Methods:**

This IRB-approved prospective feasibility study investigated patients with CPPD of the hand. All patients underwent a static 3D-CT and two dynamic 4D-CT in ulnar- and radial abduction and in supination and pronation movements to analyze instabilities of the SL region and of the distal radioulnar joint (DRUJ). Two independent readers scored the images for the presence of SL ligament and triangular fibrocartilage complex (TFCC) calcifications. Furthermore, the readers assessed the dynamic images for SL and DRUJ instabilities. Descriptive analyses were performed. Inter-rater reliability was assessed using Cohen’s kappa (κ).

**Results:**

Nine patients were included. SL ligament calcifications and instabilities were found in all patients. Of these, dynamic SL instability was detected in 77.8% of the patients, while 22.2% had a SLAC wrist. TFCC calcifications were found in 87.5% of the patients. Four patients had DRUJ instability (50%). No patient showed DRUJ instability without the presence of TFCC calcifications. Agreement between readers for calcifications was excellent (*κ* = 1) and almost perfect (*κ* = 0.89) for instabilities.

**Conclusion:**

This study provides the first evidence of relevant dynamic carpal instability in CPPD patients using advanced imaging techniques with 4D-CT, offering unique insights into wrist biomechanics.

## 1. Introduction

Calcium pyrophosphate deposition (CPPD) disease is characterized by crystal deposition in ligaments of the wrist, which can lead to interleukin-1β-mediated inflammation and damage to the ligaments. Eventually, it may result in ligament rupture with the consequence of impaired biomechanics, causing accelerated degeneration of the radiocarpal joint and intercarpal joints (so-called scapholunate advanced collapse (SLAC) wrist) (1) – similar to post-traumatic changes (2). Previous studies from our research group have demonstrated changes in collagen content of the intrinsic and extrinsic ligaments of the carpus in patients with CPPD, suggesting tissue remodeling at affected sites (3, 4). However, it remains unclear how exactly biomechanics are altered by these tissue changes and which role they play in the development of SLAC-wrist. Nonetheless, the resulting degenerative lesions plus osteoarthritis changes can lead to ongoing pain and loss of function, despite sufficient treatment of the underlying disease (5).

The advancing technical developments in the field of computed tomography (CT) open new possibilities for collecting additional information (6, 7). The most modern CT detectors allow obtaining images under motion (8, 9). This enables an excellent assessment of both static and dynamic phenomena of three-dimensional objects within a complex anatomy. The carpus is of particular interest because its complex structure escapes sufficient visualization using conventional cinematography techniques, which is especially important when recording and assessing distal radioulnar joint (DRUJ) instability (10).

The carpal structural disorders are divided into four different stages: I. ligament tears without biomechanical relevance, II. dynamic instability, III. static instability and IV. secondary osteoarthritis due to non-physiological loading (5). These are usually associated with persistent symptoms and loss of function, even with successful therapy. Surgical therapy is, therefore, usually necessary (11). Direct arthrography with an injection of contrast media into the three compartments of the carpus has served as the radiological reference standard in the assessment of carpal structural disorders. All stages of ligament rupture can be depicted with sufficient certainty. However, this procedure is invasive with a risk of complications (e.g., infection, injuries) and requires a high level of equipment and trained personnel. Initial work successfully investigated non-invasive alternatives using three-dimensional cross-sectional imaging and dynamic 4D-CT (8, 9). 4D-CT offers the advantages of conventional 3D-CT for the detection of calcifications and static (stage 3) lesions, as well as the additional ability to visualize dynamic (stage 2 lesions) (12). For this reason, 4D-CT is an established method to detect dynamic and static instabilities of the wrist (5, 8, 9, 13, 14). In addition, studies have also shown comparable results in the detection of instabilities with 4D-CT compared to arthroscopy (15).

The aim of this feasibility study was to study whether 4D-CT can be used to detect instabilities of the carpus in patients with CPPD.

## 2. Materials and methods

### 2.1. Subjects

In our prospective single-center study, we investigated patients with established diagnosis of CPPD of the hand who presented at the rheumatology in- or outpatient department of our hospital between February 2022 and February 2023. Only patients over the age of 50 being able to give written informed consent were included in this study. Patients with a history of wrist fractures, surgery or congenital deformity were excluded from the analysis. The diagnosis was made prior to study inclusion by rheumatology specialists based on clinical and imaging findings, as well as joint fluid analyses performed whenever necessary according to clinical standards.

The study was approved by the local ethics committee (EA4/004/18) and the Federal Office for Radiation Protection (Z 5–22,462/2–2018-038).

### 2.2. Imaging procedure

Before the examination, all patients were instructed extensively on how to perform the specific wrist movements. For this purpose, an instruction video was shown to each patient before the CT examination and the movement was trained. Patients were asked to move their wrist smoothly to the maximum position and then move back to the start position without abrupt movement or pause. Two 4D-CT scans were performed: (1) for SL ligament instability assessment, the examination was performed in ulnar and radial abduction, and (2) for DRUJ instability assessment, the examination was performed in supination and pronation. To reduce deviations in movement performance, the video was also played during the scan. In all patients, the clinical dominant hand (according to tenderness and/or swelling) was scanned. The examination was performed while the patient was standing beside the CT scanner, with the hand to be examined positioned into the isocenter of the gantry.

**Figure 1:**
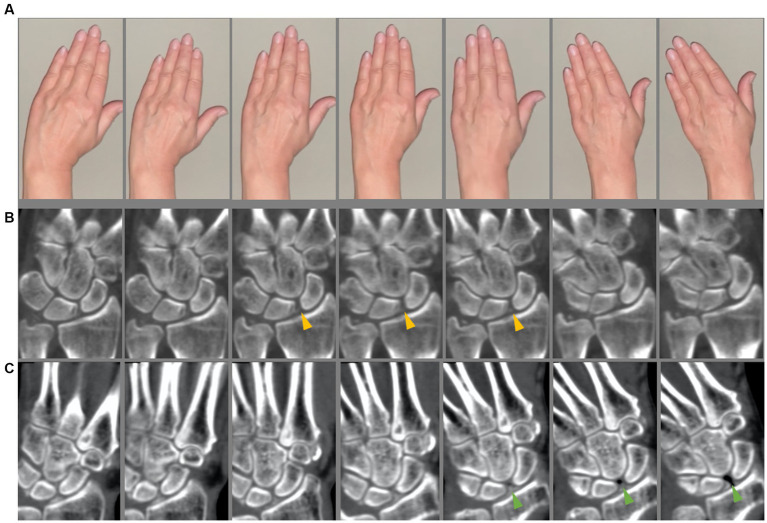
4D-CT scan. **(A)** Sequences of the instruction video. **(B,C)** Four-dimensional computed tomography scan in two different calcium pyrophosphate deposition disease (CPPD) patients. **(A,B)** Slight scapho-lunate (SL) dissociation is seen (yellow arrow heads) indicating a dynamic SL ligament instability. **(C)** CPPD patient with clearly SL dissociation with vacuum effect (green arrow heads).

**Figure 2:**
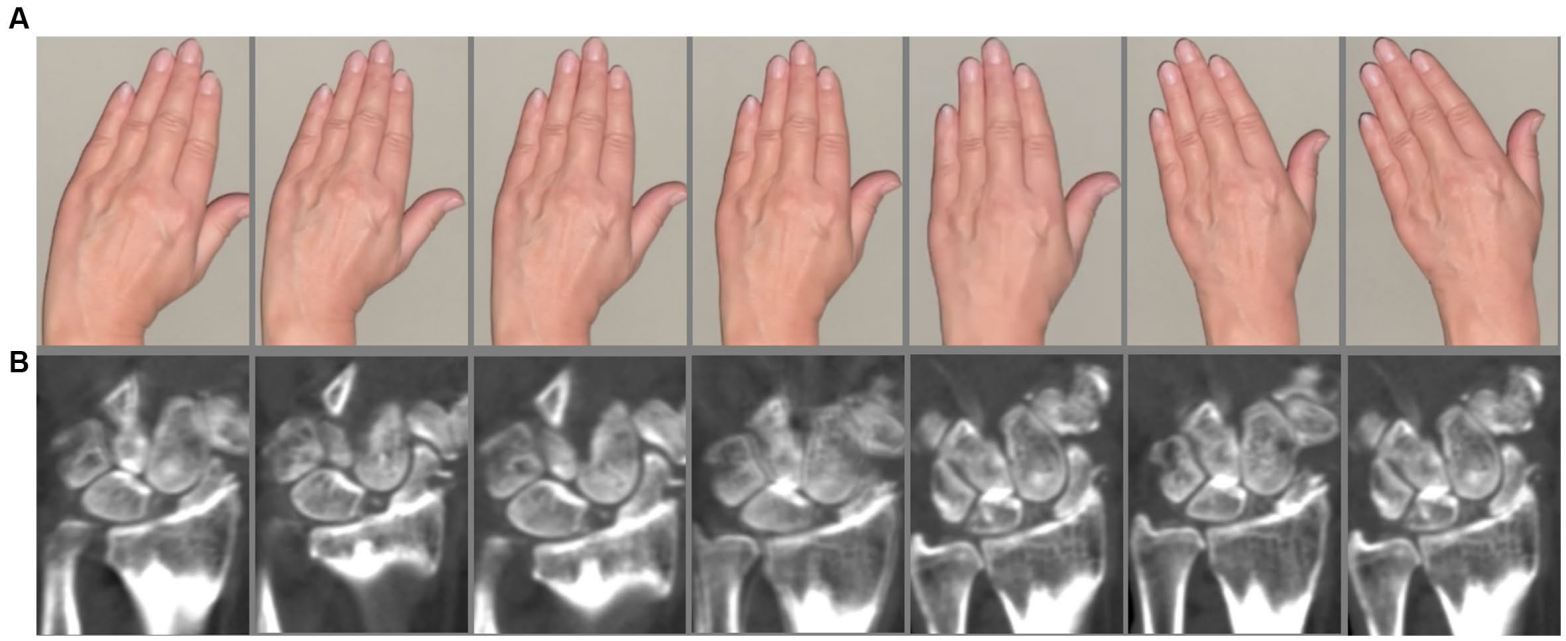
4D-CT scan. **(A)** Sequences of the instruction video. **(B)** Patient with SLAC wrist. Static SL ligament instability with severe osteoarthritis.

First, a static 3D-CT of the wrist was acquired. 4D-CT was performed on a 320-row single-source CT scanner (Canon Aquilion ONE Vision, Canon Medical Systems, Otawara, Japan) in continuous volume mode with 16 cm z-axis coverage without table movement. Rotation time was 0.275 s. The scans were performed using 80 kVp and 13.75 mAs. The dynamic scans were performed continuously for 8 s. For each wrist movement, 33 volume series were reconstructed (image examples are presented in Figures 1, 2) with 0.5 mm slice thickness using a medium soft tissue kernel. Static images were reconstructed as 0.5 mm multiplanar reconstructions in a medium soft tissue and sharp bone kernel. The radiation exposure (estimated effective dose) was calculated using the overall dose-length product (DLP) and a conversion coefficient of 0.0008 [mSv x mGy^−1^ x cm^−1^] (16, 17).

### 2.3. 4D-CT image processing

All images were pseudonymized for name, age, and sex. The dynamic CT reconstructions were performed on the Visage Imaging Workstation (Visage 7, Visage Imaging, Inc., San Diego, CA, United States). This was performed separately to analyze SL instability in coronal planes and DRUJ instability in transversal planes.

### 2.4. Image reading

Two trained independent radiologists (S.T.U. /K.Z. with three/seven years of experience in musculoskeletal imaging) scored the 4D-CT images for the presence of calcifications of the SL and LT ligaments as well as of the triangular fibrocartilage complex (TFCC). Wrist instabilities were evaluated using the 4D-CT datasets with the following categories for SL instability: (0) no SL instability, (I) dynamic SL instability (SL distance greater than 3 mm only during dynamic examination), (II) static SL instability (SL distance greater than 3 mm in static examination) and (III) SLAC-wrist. In a second scoring session, the DRUJ instability was assessed which was defined as dynamic dorsal subluxation of the distal ulna. The readers were blinded to all identifying and clinical information. Cases of disagreement were solved after the reading of an expert musculoskeletal radiologist with 13 years of experience (T.D.).

### 2.5. Statistical analysis

Statistical analysis was performed using GraphPad Prism (Version 7 for MacOS, GraphPad Software, La Jolla, California, United States). Descriptive statistics were calculated to assess frequencies of calcifications and wrist instabilities. Inter-rater reliability was assessed using Cohen’s kappa (*κ*) (18) for the presence or absence of calcifications and wrist instabilities, separately.

## 3. Results

### 3.1. Subjects

Nine patients (seven women) with CPPD were included in the study. In one patient, the DRUJ instability and the calcification of the TFCC could not be assessed because of surgery of the distal ulna. The patients had a mean age of 77 years (SD 6.1, range 64–85 years) with a mean duration of CPPD diagnosis of 11.4 years (SD 8.7). At the time of the study inclusion, seven of nine patients suffered from wrist pain. Only one patient received physical therapy of the wrist in the last 2 years. Total DLP was 474 mGy*cm with an estimated effective dose of 0.379 mSv.

### 3.2. Image reading and statistical analysis

All patients showed calcification of the SL ligament (100%). In seven of nine patients, the LT ligament was calcified (77.8%). In seven of eight patients, calcifications of the TFCC were found (87.5%). SL instability was found in all patients: Of these, seven of nine patients had dynamic SL instability (77.8%), and two of nine patients had a SLAC wrist (22.2%). No patients showed static SL instability. DRUJ instability was shown in four of eight patients (50%). No patient showed DRUJ instability without the presence of TFCC calcifications. Both patients without wrist pain at the time of the study inclusion showed only dynamic SL instability without DRUJ instability. Agreement between readers for calcification was excellent with *κ* = 1. Only in one case of SL instability the readers have a disagreement resulting in an almost perfect agreement for instabilities with *κ* = 0.89.

## 4. Discussion

To the best of our knowledge, this is the first study investigating the feasibility of four-dimensional CT in the detection of carpal instabilities in patients with CPPD disease. In addition to CPPD crystal deposits, all patients also showed wrist instabilities. This was especially pronounced for SL ligament instability, which was observed in 77.8 and 22.2% were even exhibiting final stages of the so-called SLAC wrist. Half of the patients investigated also had instability of the DRUJ.

The knowledge of these biomechanical processes is of great importance in preventing or delaying the development of irreversible destruction. Previous studies from our research group have shown that there is remodeling processes of the intrinsic and extrinsic ligaments of the carpus in patients with CPPD (3, 19). However, these changes in the intrinsic and extrinsic ligamentous structures of the wrist may have a decisive influence on the biomechanics of the carpus with development of wrist instabilities.

Crystal depositions in patients with CPPD disease are especially localized in the region of the TFCC. Concomitantly, the TFCC, with its disc-like structure, serves as the main stabilizer of the DRUJ. Our results allow us to assume that micro- and macroscopic changes are present here, which lead to an altered biomechanical property (20) and thus contribute to instabilities in the DRUJ. These changes can subsequently lead to different biomechanical properties of the tissues, a change in force distribution and degeneration of the wrist components. Furthermore, it is worth considering that possible lesions related to IL-1 release may also occur apart from the inflammatory attacks of the disease, the influence of which may play a role in the development of degenerative changes. The carpus is of particular interest for 4D-CT because certain motion patterns cannot be accurately visualized with conventional cinematographic techniques or with sonography (in cases of DRUJ instability). This is particularly important for visualization and assessment of DRUJ instability.

This investigation was deliberately designed as a feasibility study with a small sample size. This leads to a lack of statistical power and the results should therefore be taken with caution. We also did not collect information about the extent of the calcification which may have the greatest impact in developing wrist instabilities. The assessment was performed qualitatively, further research using quantitative methods in a greater number of investigated patients is therefore needed to further understand the altered biomechanics in wrist instabilities. However, the essential limitation of our study is, that we did not include healthy, age-matched subjects in our feasibility study. Furthermore, deeper insights will be gained from comparisons with individuals with other types of joint disease, such as rheumatoid arthritis.

Our study gives first evidence that 4D-CT allows for unique insights into the multidimensional dynamic biomechanics of the wrist in patients with CPPD disease. The assessment of these processes in CPPD has not been researched so far and therefore opens up a broad spectrum not only in understanding the pathophysiological processes but also in the potential translation in new therapeutic approaches. In the course of the disease, these ligament tears result in secondary osteoarthritis, which can cause pain and persisting symptoms, even without active inflammation from the underlying disease. In the future, dynamic imaging may be used to better differentiate between patients with predominantly mechanic and inflammatory disease courses to avoid unnecessary therapy.

## Data availability statement

The raw data supporting the conclusions of this article will be made available by the authors, without undue reservation.

## Ethics statement

The studies involving humans were approved by the local ethics committee (EA4/004/18). The studies were conducted in accordance with the local legislation and institutional requirements. The participants provided their written informed consent to participate in this study.

## Author contributions

SU, KZ, and TD: conceptualization. SU and TD: methodology, software, and funding acquisition. SU and KZ: validation. SU: formal analysis, resources, writing – original draft preparation, and visualization. SU, KZ, LP, SO, and TD: investigation and writing – review and editing. SU and LP: data curation. TD: supervision. KZ and TD: project administration. All authors have read and agreed to the published version of the manuscript.

## Funding

This work was funded by grants from the German Society of Musculoskeletal Radiology (Deutsche Gesellschaft für muskuloskelettale Radiologie; DGMSR). SU is participant in the BIH-Charité Junior Digital Clinician Scientist Program funded by the Charité – Universitätsmedizin Berlin and the Berlin Institute of Health. TD reports funding from the Berlin Institute of Health (BIH) during the conduct of this study. KZ reports funding (research grant) from the Assessment of Spondyloarthritis international Society (ASAS). LP and SO report no funding. We acknowledge financial support from the Open Access Publication Fund of Charité - Universitätsmedizin Berlin and the German Research Foundation (DFG).

## Conflict of interest

The authors declare that the research was conducted in the absence of any commercial or financial relationships that could be construed as a potential conflict of interest.

## Publisher’s note

All claims expressed in this article are solely those of the authors and do not necessarily represent those of their affiliated organizations, or those of the publisher, the editors and the reviewers. Any product that may be evaluated in this article, or claim that may be made by its manufacturer, is not guaranteed or endorsed by the publisher.
